# Is Poly(methyl methacrylate) (PMMA) a Suitable Substrate for ALD?: A Review

**DOI:** 10.3390/polym13081346

**Published:** 2021-04-20

**Authors:** Marta Adriana Forte, Ricardo Manuel Silva, Carlos José Tavares, Rui Ferreira e Silva

**Affiliations:** 1 CF-UM-UP, Centre of Physics of Minho and Porto Universities, Campus of Azurém, University of Minho, 4800-058 Guimarães, Portugal; martadrianaff@gmail.com (M.A.F.); ctavares@fisica.uminho.pt (C.J.T.); 2 CICECO, Department of Materials and Ceramic Engineering, University of Aveiro, 3810-193 Aveiro, Portugal; rmsilva@ua.pt

**Keywords:** poly (methyl methacrylate) (PMMA), atomic layer deposition (ALD), polymeric substrate, metal oxide, thin films

## Abstract

Poly (methyl methacrylate) (PMMA) is a thermoplastic synthetic polymer, which displays superior characteristics such as transparency, good tensile strength, and processability. Its performance can be improved by surface engineering via the use of functionalized thin film coatings, resulting in its versatility across a host of applications including, energy harvesting, dielectric layers and water purification. Modification of the PMMA surface can be achieved by atomic layer deposition (ALD), a vapor-phase, chemical deposition technique, which permits atomic-level control. However, PMMA presents a challenge for ALD due to its lack of active surface sites, necessary for gas precursor reaction, nucleation, and subsequent growth. The purpose of this review is to discuss the research related to the employment of PMMA as either a substrate, support, or masking layer over a range of ALD thin film growth techniques, namely, thermal, plasma-enhanced, and area-selective atomic layer deposition. It also highlights applications in the selected fields of flexible electronics, biomaterials, sensing, and photocatalysis, and underscores relevant characterization techniques. Further, it concludes with a prospective view of the role of ALD in PMMA processing.

## 1. Introduction

Poly (methyl methacrylate) (PMMA) is a transparent thermoplastic synthesized by emulsion polymerization, solution polymerization, and bulk polymerization from the MMA monomer [1]. This acrylate has high resistance to sunlight exposure and good optical properties, widely used to substitute and enhance the glass performance [2]. This polymeric compound is attractive; hence is stable, affordable, has been explored in multiple structural and forms—like sheets, films, tubular, even spherical composites—from nanotechnology to upper metrics/scales with variety being applied in all kinds of industries [3,4]. One of the main advantages of PMMA is that it contains less potentially harmful subunits from the synthesis, like bisphenol-A, commonly found in other types of polymers such as polycarbonates, polysulfones, and epoxy resins [5]. It is a superior polymeric material for analytical separation, sensing [6], biomedical and medical applications due to biocompatibility [7,8], and is used for electrolysis [9], polymer conductivity, viscosity measurements [10,11], solar nano/micro concentrator lens for solar cells [12,13,14]. In practice, the PMMA surface properties can be tailored by surface modification through graft copolymerization [15] or by the incorporation of a surfactant into the polymer matrix [16].

As an alternative to the previous approaches, the use of atomic layer deposition (ALD) is among the most promising research lines for PMMA surface engineering. Additionally, ALD offers the possibility of operating at low deposition temperatures making it highly compatible not only with the PMMA but also with other polymers. As matter of fact, the potential of ALD to prepare and modify nanomaterials is well recognized, and a significant number of excellent reviews are available for both types of objectives, in particular, surface engineering of high surface-area nanostructures [17,18].

ALD is a variant of chemical vapor deposition (CVD) technique, where two or more gas precursors are pulsed, separately, into the reaction chamber, under controlled pressure. Each one has a correspondent chemical reaction and the process repeats sequentially, the thickness of the coating increasing with the number of repetitions (cycles) [19,20,21,22,23]. For example, in the CVD operating principle for metal oxides, the metal precursor and water are kept in two separated stainless-steel reservoirs and then pulsed into the reaction chamber in a sequential mode through an inert carrier gas (e.g., Ar, N_2_) flow. In opposition to CVD, in ALD there are no gas-phase reactions, in this way, the thickness is simply controlled by the number of cycles. The resulting thin films are conformal and uniform, with just a single monolayer per cycle [21,24,25].

**Figure 1:**
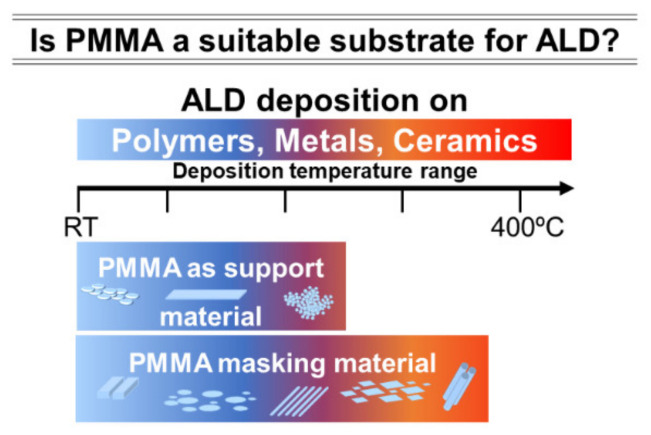
Schematic illustration of ALD application range: deposition temperature and types of substrates. The ability to perform ALD at low temperatures is well suitable to deal with thermally sensitive materials such as PMMA. As a support material, PMMA can be produced such as thick plates, spin-coated films, powder, nanoparticles, amongst other forms.

Comparing with other deposition techniques, ALD has a low deposition rate; hence the time required for coating is longer, where the viability for industrialization is a concern [23,26]. Despite this disadvantage, ALD can operate at low temperatures, as mentioned above; notably, the coatings have high-quality and purity with the absence of voids or pinholes [26,27]. Thus, a versatility of substrates in morphology and composition emerged, including polymeric based compounds (Figure 1) [19,20,21,22]. For example, an inorganic thin film with a few atoms or nanometers in thickness on PMMA can drastically change its surface properties.

This article is a review focused on ALD studies aiming at PMMA surface modification with metal or metal oxide coatings, where PMMA is used as substrate as well as masking layer (e.g., self-assembled monolayers, SAM) in nanofabrication-based patterning methods. The deposition of these coatings is associated with several subcategories of ALD, such as thermal atomic layer deposition (T-ALD), plasma atomic layer deposition (PE-ALD), and area-selective atomic layer deposition (AS-ALD), also referred to as selective-area ALD [17].

## 2. Brief History of Atomic Layer Deposition

Atomic layer deposition discovery took place in Europe, in two different countries separated by a few years. The first evidence of ALD was in the 1960s in USSR, by Aleskovskii and Koltsov (1965) [28], Shevjakov et al. (1967) [29] and Sveshnikova et al. (1969) [30], and they called it molecular layering. The works mentioned the deposition of metallic compounds on silicon surfaces. After a few years, not so far away, Tuomo Suntola developed the atomic layer epitaxy (ALE) process where ZnS, SnO_2_, and GaP were coated for electroluminescent flat panel displays. This work was patented and expanded not only in Finland but also in other countries [31,32]. The ALE experiments were very directed to halides in a gas–solid system, and in the 1970s started the depositions of other chemical elements [22]. In 1972 the first implementation of polymeric foils as a substrate was used by Suntola to create a miniaturized device able to measure the humidity in solid-state. This study resulted in a patent and nowadays these devices are very used regarding efficiency [33].

The 1980s were remarkable for this technology. The number of ALD publications was increasing, and alkyls and β-diketonates were used as reactants to generate new deposition processes for semiconductors [22]. Because of great interest in ALD research topic, the first conference about this expertise was organized in 1984. In parallel, ALD industrialization for electroluminescent displays took place with the name Lohja’ s [34]. ALE started to be applied in other fields, such as solar cells, catalysis, and microelectrochemistry industries. Regarding the application on polymer surfaces, in 1984 Motsenyat et al. prepared polyamide substrates coated with titanium oxide (TiO_2_) [35].

In 1990, Markku Leskelä suggested changing the name ALE to ALD, at the “International Symposium on Atomic Layer Epitaxy” [20,34]. As a result, there was an increment of inorganic reactants based in cyclopentadienyls, alkoxides, and alkylamides, to produce binary systems (e.g., metal oxides) [22], as well as the development of ternary systems [34]. Alumina (Al_2_O_3_) was (and is) a well-established coating process, however, the growth is amorphous instead of epitaxial, so that was the driving force to change the name and amplify the variety of substrates [36,37].

Currently, ALD is mostly applied in microelectronics field, such as transistors, capacitors, energy storage, conversion, biomimetic membranes, and graphene for desalination supports, catalysts, and medical applications [38]. A very actual topic is the ALD modification of soft materials’ surfaces, especially thermally fragile polymers, which are very challenging to processing due to the low deposition temperatures required or pre-functionalization treatments, as deeply presented in the following.

## 3. Coatings on PMMA by Thermal Atomic Layer Deposition

### 3.1. PMMA Challenges for ALD

**Figure 2:**
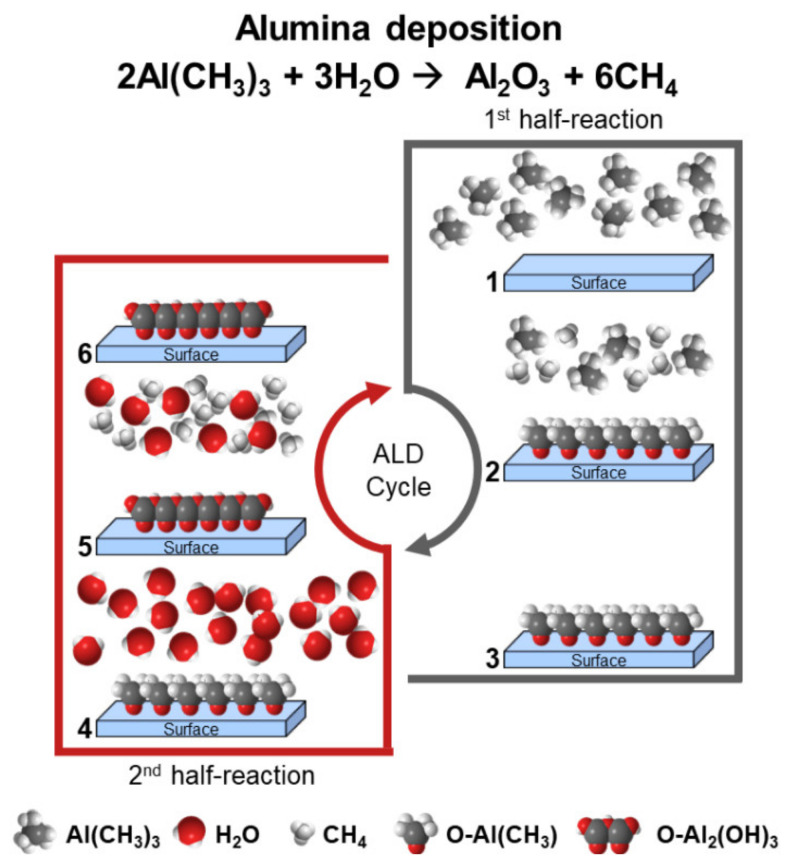
Schematic representation of the ALD formation of the first Al_2_O_3_ monolayer from: (1) TMA pulsing; (2) TMA chemisorption in the surface (first half-reaction); (3) after purging of unreacted TMA and methane; (4) water pulsing; (5) water chemisorption on the by-product from the first half-reaction; and (6) after purging of unreacted water and methane.

PMMA surface engineering opens new opportunities to modify the polymer surface chemistry to attain improved properties with a second material. The ability to control the reaction between the ALD precursors and the PMMA surface paves the way for the ALD processing. From a practical point of view, the ALD coating is a product from sequential self-limiting surface reactions of two or more precursors, which make up an ALD cycle (Figure 2). In this context, inorganic compounds such as binary or ternary metal oxides can be produced depending on the number of precursors in the ALD process [27].

The mechanism of ALD on polymeric substrates, for binary reactions, is constituted by A and B precursors, that will react by chemisorption and create a solid AB coating (e.g., metal oxide) [19,39,40]. Precursor A is the metal source, and precursor B is the non-metal, such as H_2_O or O_3_. In the example illustrated in Figure 2 the first precursor, trimethylaluminium, (Al(CH_3_)_2_/TMA) is introduced in the chamber and reacts on the surface, followed by its diffusion; this step is named as half-cycle, and the final product is named ligand or by-product [19,40]. Then, the second precursor, water, is pulsed and reacts with the resulting previous ligands [19,40]. It is the complementary half cycle, together with the previous, that makes a cycle with the desirable O-Al_2_(OH)_3_ monolayer. With the repetition of these two half-cycles, the Al_2_O_3_ growth takes place. It is worth to mention that between the two half-reactions there is a purging step assisted by the introduction of inert gas (e.g., Ar or N_2_). A homogeneous growth is promoted, preventing precursor–precursor, precursor–by-product, by-product–by-product reactions [19]. Consequently, the formed coatings are very precise and controlled in a conformal configuration [41].

**Figure 3:**
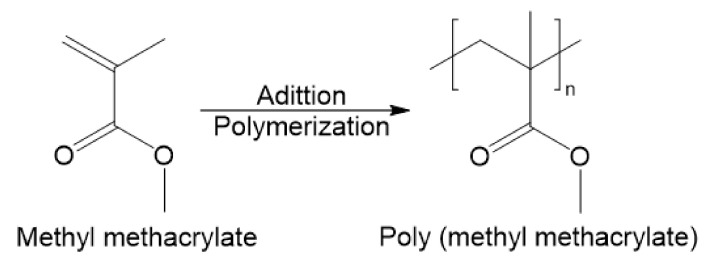
Synthesis of PMMA by addition polymerization of MMA (adapted from [1]).

The PMMA polymer is a very stable material; in other words, inert, consisting in strong chemical bonds, which hinder the modification of the chain [42]. The ester’s presence, R-COOR’ in Figure 3, raises the polarity and limits the ALD coating because, generally, the precursors are nonpolar [40].

**Figure 4:**
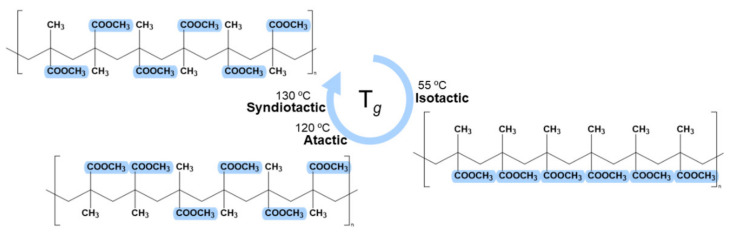
PMMA tacticities: isotactic, syndiotactic, and atactic and respective glass transition temperature. The tacticities values were collected from [44].

A second challenge is the low transition temperature value (T*_g_*) of PMMA. This polymer presents three main tacticities (isotactic, syndiotactic, and atactic), where pendant groups or hydrogens are laid in certain positions. In Figure 4, it is possible to observe these structures [43]. The chiral central’s orientation will determine the transition temperature and crystallinity, thermal resistance, solubility, degree of biocompatibility, hydrolyzation, and other properties. For example, the T*_g_* is the lowest (55 °C) for the isotactic structure when ester groups are disposed on one single side of the backbone structure, from a random or regular order, respectively. The chemical structure is more stable for the atactic and syndiotactic structures, so automatically increasing the T*_g_* to 120 and 130 °C, respectively. The different percentages of tacticities result in an almost specific T*_g_* for each PMMA substrate [44].

Depending on the ALD process, the deposition temperature ranges between room temperature (≈20 °C) and 400 °C. In this context, the deposition temperature should be below the T*_g_* of the PMMA to ensure that the polymer remains in the solid-state. For example, if the deposition temperature is too high, PMMA will start to decompose [40,45,46,47,48,49,50,51]. Considering that the majority of ALD processes occur for temperatures >100 °C, the PMMA T*_g_* value plays a key factor in the selection of the ALD process. One of the most investigated precursor combinations in ALD for Al_2_O_3_ deposition is TMA with H_2_O and the deposition temperature is typically ≤300 °C. Al_2_O_3_ gives an example of a material that tends to grows in an amorphous form and it has been applied to thermally fragile substrates [17].

### 3.2. Nucleation and Growth Studies

**Figure 5:**
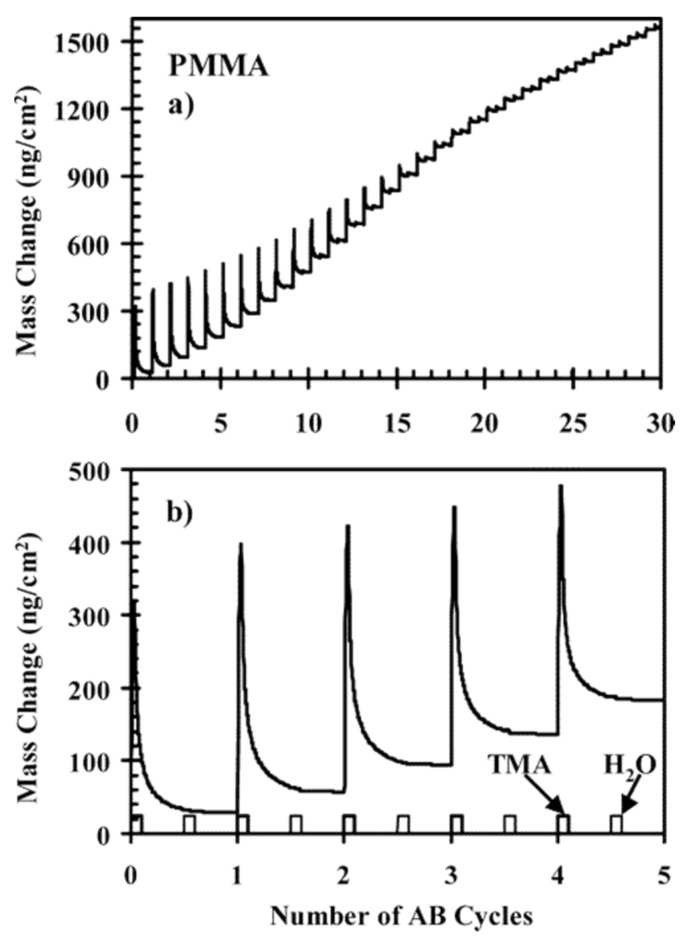
Mass change measurements by QCM versus number cycles of Al_2_O_3_ ALD on PMMA at 85 °C for (**a**) 30 and (**b**) 5 cycles (reprinted with permission from [40], Copyright (2005) American Chemical Society).

It can be found in the literature reports providing insights into ALD process and mechanism of Al_2_O_3_ growth on PMMA. For instance, Wilson et al. studied the nucleation and growth of Al_2_O_3_ on spin-coated thin films of various polymeric compounds, including PMMA. To this end, the nucleation and growth process kinetics were monitored by the mass changes measured by a quartz crystal microbalance (QCM). In the first five cycles, TMA is adsorbed. Due to the insolubility of PMMA in the Al_2_O_3_ precursor, 90% of TMA is desorbed when the water is pulsed. The remaining 10% are then hydrolyzed. With the increasing cycles, there are more functional groups from adsorbed TMA, and the desorption decreases. After 20 cycles, the desorption is almost negligible (Figure 5) [40].

**Figure 6:**
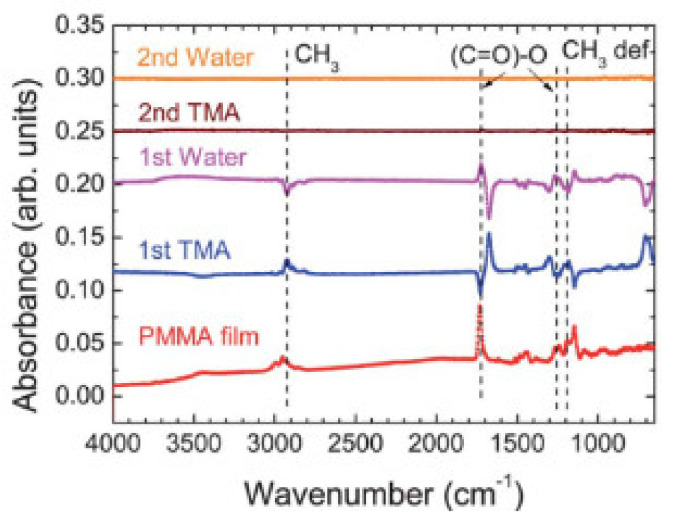
In-situ FTIR analysis of uncoated PMMA spin-coated onto Si, first, and second ALD half-reactions. Al_2_O_3_ coating was made from TMA and H_2_O at 80 °C. A FTIR run followed each half-cycle, and between half-cycles, the reactor was purged for 10 s (republished with permission of Be Gong, from [52]; permission conveyed through Copyright Clearance Center, Inc.).

The Al_2_O_3_ deposition mechanism on a ~200 nm thick PMMA film was also studied by in-situ Fourier transform infrared spectroscopy (FTIR). This study was carried out in an adapted ALD chamber to run the FTIR measurements, allowing the analysis of each ALD half-cycle to understand the correspondent reaction [52]. The FTIR results presented in Figure 6 suggest that the aluminum attacks the ester, which decreases the amount of C=O and C-O FTIR bands; the product from the first half-reaction being aluminum carbonate. Then, as H_2_O is pulsed into the chamber, FTIR reveals a decrease in -CH_3_ groups, which corresponds to the Al-CH_3_ and aluminum carbonate removing [52]. These studies demonstrate that a strong correlation exists in the PMMA surface chemistry to initiate Al_2_O_3_ by ALD. Moreover, the propensity for TMA or other ALD precursors to react on the surface or to diffuse in the sub-surface depends on the polymer [40,52].

### 3.3. Adhesion and Mechanical Properties

**Figure 7:**
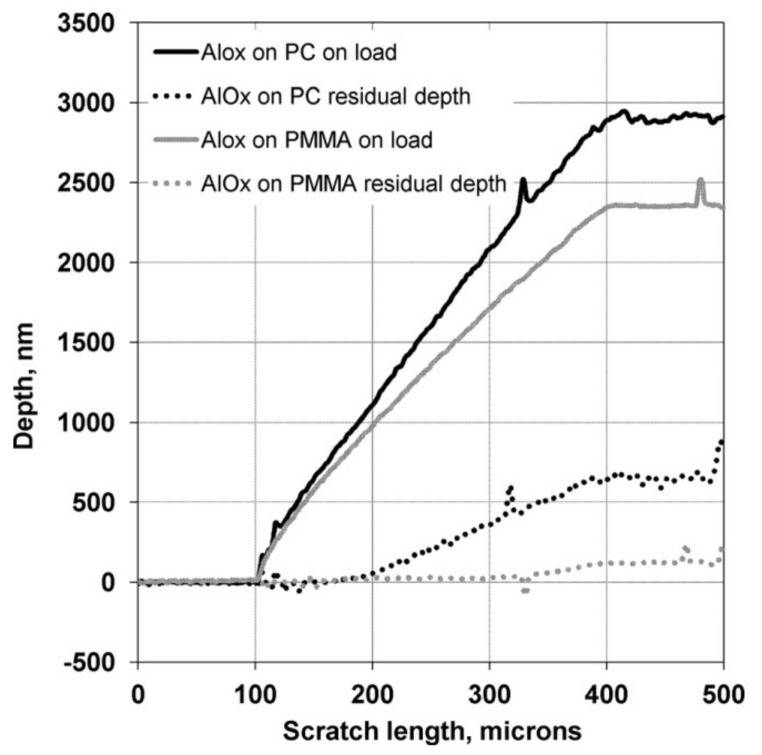
On-load and residual depth curves for Al_2_O_3_/PMMA and Al_2_O_3_/PC (polycarbonate) after the nanoscratch. The test was repeated five times per sample; each scratch had 500 µm of length, at a scanning velocity of 10 µm/s, and a ramped load varying from 0.1 mn to 60 mn (reprinted with permission from [53], Copyright 2012, American Vacuum Society).

The possibility of coating polymeric surfaces with a thin film of a metal oxide opened a wide range of potential applications. However, besides the T*_g_* temperature and the surface polarity issues, the thermal and mechanical properties of the polymers may limit ALD coating. Here, the mechanical and tribological properties of Al_2_O_3_ ALD thin films on PMMA plates were studied by nanoscratch testing, where the coating/substrate system is comprised by ‘hard’ coatings on ‘soft’ flexible PMMA substrates. Prior to the Al_2_O_3_ deposition by ALD, the PMMA plates were pre-cleaned with a 5% sodium hydroxide solution for a short period of time and then in de-ionized water ultrasonic bath at room conditions to ensure an impurity-free substrate. An 85 nm thick Al_2_O_3_ film on PMMA plate was submitted to nano-scratch tests through a diamond spherical indenter with 25 µm of radius. The results suggest that there is a net elastic recuperation when the load is eliminated, and the Al_2_O_3_/PMMA resisted to plastic deformation up to 340 µm of scratch length, presenting a residual depth (~0.05 µm) at the end of the test, as illustrated in Figure 7.

In parallel to Al_2_O_3_/PMMA, an Al_2_O_3_/PC (polycarbonate) system was also characterized under the same conditions (Figure 7) and the obtained results for both systems are directly related to the intrinsic properties of the polymeric substrates. In this way, the Young’s modulus and hardness of PMMA are higher than that of PC and will therefore give more support to the hard film and a higher scratch resistance [53]. It is noteworthy that there was no coating delamination was observed in both systems.

**Figure 8:**
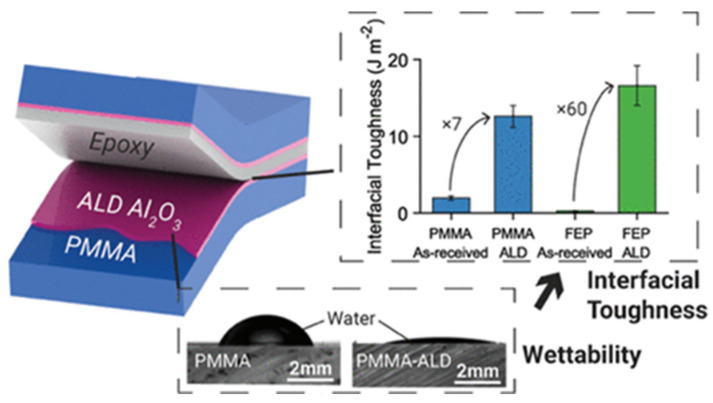
Thermoplastic-epoxy resin with the double Al_2_O_3_ coating; wettability test of a water droplet in coated and uncoated PMMA surface, and the interfacial toughness of PMMA and fluorinated ethylene propylene (FED) (wedge test) (reprinted with permission from [54], Copyright (2019), American Chemical Society).

Concerning the problematics of adhesion and mechanical resistance, Chen et al. reported on the enhancement of the PMMA and epoxy resin’s interfacial toughness by depositing Al_2_O_3_ thin films at 65 °C onto PMMA surface, to avoid fracture and delamination of polymer interfaces, as shown in Figure 8 [54]. This interfacial improvement is particularly important in applications such as fiber reinforced composites, flexible electronics, and encapsulation layers for photovoltaics where the adhesion between two substrates is crucial. The role played by Al_2_O_3_ ALD (130 nm thick) on PMMA surface was the wettability modification assessed by the decrease of the water contact angle (WCA) and consequently the increase of the polymer surface energy. It is of great importance to understanding this property in the adhesion phenomena which relates to physicochemical properties of the surface as well as with the mechanical properties. This work also emphasized the versatility of ALD in engineering the adhesive properties of chemically inert polymer surfaces.

Shahmohammadi et al. also studied the adhesion and mechanical properties. This group used TDMAT and ozone to deposit a TiO_2_ thin film onto PMMA with excellent properties without plasma assistance or seed layers. Taking into account the PMMA thermal stability (120 °C), the ALD reactor temperature was established by thermogravimetric analysis. The samples were coated from 50 to 500 cycles to understand the PMMA thickness, and the optimized growth per cycle was 1.39 Å/cycle. The presence of titanium was confirmed by X-ray photoelectron spectroscopy (XPS) and X-ray absorption near-edge structure (XANES). The coated PMMA reduced the water contact angle from 84° to almost 20°, which means a remarkable hydrophilicity improvement. The hardness of the sample with 30 nm TiO_2_ was tested by Vickers’ hardness method, applying a 300 g-force; the results were improved by almost 60% [55].

### 3.4. Applications

#### 3.4.1. Applications in Photonics, Photoluminescence, and Photocatalysis

**Figure 9:**
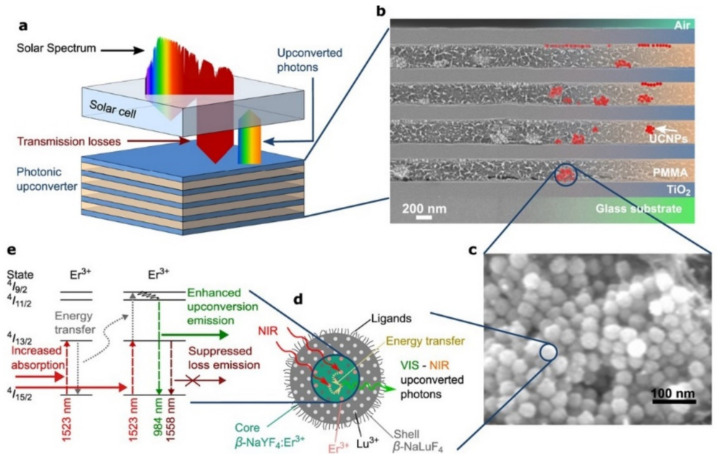
(**a**) Bragg stack (multilayered material comprised of TiO_2_ layers/PMMA+NPs) for charge generation in a solar cell by a photonic upconverter. SEM image of the (**b**) Bragg stack and (**c**) of upconverter nanoparticles. (**d**) Scheme of core–shell upconverter NPs, converting near-infrared (NIR) to visible (VIS) photons in the core. (**e**) Energy levels in the upconverter Er^3+^ and the upconversion process [from [57] this work by C.L.M. Hofman is licensed under a Creative Commons Attribution (CC BY 4.0)].

In another study, Hofmann et al. reported that thin layers of TiO_2_ by ALD were used in the fabrication of an organic-inorganic hybrid Bragg stack and the photonic effects of the as-prepared Bragg stack were investigated on upconversion luminescence [56,57]. The architecture of the Bragg stack consists of TiO_2_ ALD layers and PMMA with sodium yttrium fluoride active nanoparticles (NPs) doped with trivalent erbium ions (β-NaYF_4_:25%Er^+3^), mixed on borofloat 33 glass. The PMMA layers containing the active nanoparticles were produced by spin-coating. The number of stacked layers and their thickness plays a major role in the refractive indexes on upconversion luminescence performance and the low-temperature ALD process (100 °C) of TiO_2_ from the reaction between titanium tetrachloride (TiCl_4_) and H_2_O revealed compatibility with the PMMA based Bragg stack multilayer material (Figure 9).

Apart from Al_2_O_3_ and TiO_2_ ALD coatings, PMMA has been used as a support for crystalline zinc oxide (ZnO). Sing et al. reported on the coating of various thicknesses of PMMA thin films (5 nm, 32 nm, and 80 nm) spin-coated onto silicon (Si) substrates. The ZnO photoluminescence activity was evaluated as a function of the underneath PMMA film thickness, ZnO structure and morphology. The as-deposited wurtzite ZnO presented a strong orientation along the c-axis which is critical to the photoluminescence activity enhancement as well as reduction in thickness of PMMA templates. Interestingly, the ZnO deposition was carried out at near ambient temperature (ca. 35 °C) from diethylzinc, (Zn(C_2_H_5_)_2_/DEZ) and H_2_O [58].

**Figure 10:**
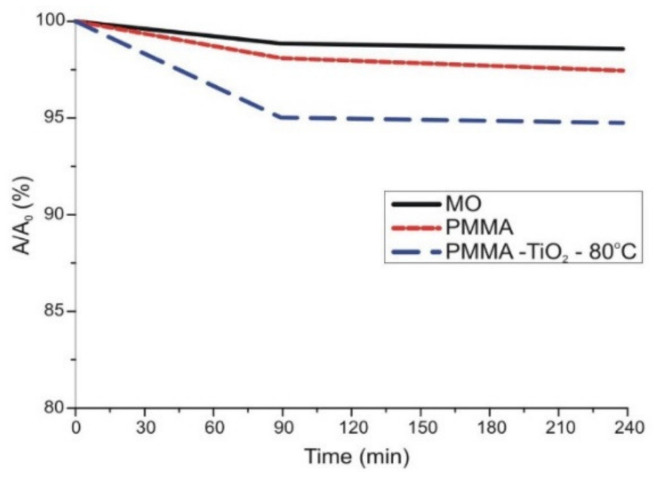
Photocatalytic efficiency of the amorphous TiO_2_. After 90 min, the methylene orange dye degraded 5% under UV-A illumination [adapted from [59], this work by Orsolya Kéri is licensed under a Creative Commons Attribution (CC BY 4.0)].

PMMA has also been coated by ALD to develop photocatalysts. One of the key materials in the degradation of organic pollutants is TiO_2_, which presents photocatalytic activity. Kéri et al. investigated the photocatalyst activity of ALD grown TiO_2_ deposited at 80 °C onto PMMA nanoparticles (50–100 nm) prepared by emulsion polymerization. As a result, amorphous TiO_2_ was obtained with detectable photocatalytic effect under UV-A illumination (Figure 10). This was relatively unexpected for an amorphous phase, once it is well known that the crystalline anatase TiO_2_ polymorph presents the highest photocatalytic activity. The authors suggested that this effect may be related to the C incorporation during the ALD deposition. This statement was based on the increment of the C 1 s signal from the X-ray photoelectron spectroscopy (XPS), when compared with the same film produced by sputtering [59].

**Figure 11:**
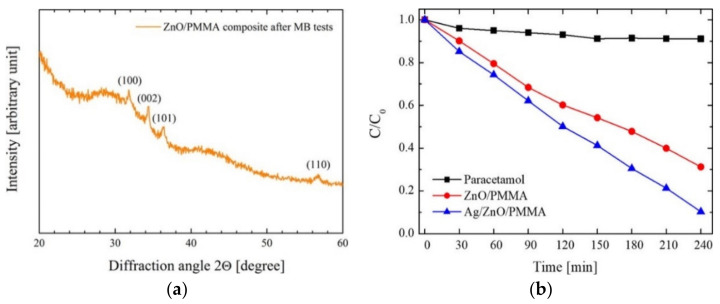
XRD pattern of ZnO/PMMA composite after the seven MB discoloration runs [62]. (**a**) Degradation of paracetamol drug as a function of irradiation time for paracetamol alone (squares), paracetamol with ZnO/PMMA (circles), and paracetamol with Ag/ZnO/PMMA (triangles) samples (**b**), under UV illumination [61] (both works by Alessandro Di Mauro are licensed under a Creative Commons Attribution (CC BY 4.0)).

ALD of ZnO was also studied on PMMA in the elaboration of photocatalysts for wastewater treatment. Di Mauro et al. explored ZnO/PMMA and Ag/ZnO/PMMA nanocomposites as photocatalyst materials for the degradation of pollutants and water reuse upon UV light illumination [60,61,62]. The authors used different forms of PMMA i.e., commercial PMMA powders (0.2–1 mm in diameter) and plates of PMMA (4 mm thick), and kept the deposition temperature at 80 °C, a temperature compatible with the thermal stability of the PMMA polymer which enabled the growth of polycrystalline ZnO wurtzite [60,61,62]. After the photocatalytic degradation tests, where an organic dye (methylene blue) was used as a model pollutant, the ZnO on the PMMA nanocomposites demonstrated excellent photo-stability [62]. The addition of Ag to the ZnO/PMMA nanocomposites brought new features to the nanocomposite, in terms of improving the photocatalytic efficiency allowing degradation tests of other organic contaminants (e.g., paracetamol drug, sodium lauryl sulfate), besides methylene blue [61]. Figure 11a shows the X-ray diffraction (XRD) patterns of the ZnO/PMMA composite after the degradation tests, where it is possible to observe that ZnO remained stable, maintaining the wurtzite crystallographic structure [62]. On the other hand, Figure 11b reveals the degradation of pharmaceuticals considered as emerging contaminants by the Ag/ZnO/PMMA nanocomposite, under UV illumination [61]. In summary, these nanocomposites were stable and reusable in the degradation of organic pollutants and are easy to prepare and to recover after being tested.

#### 3.4.2. Applications in Dentistry

**Figure 12:**
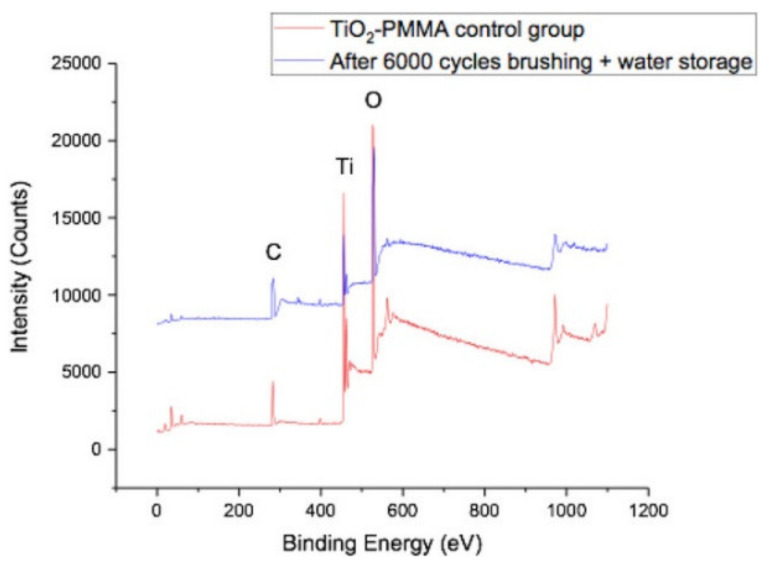
XPS results of coated PMMA unbrushed (red) and brushed (blue), storage in water and brushed again after 5 months [48]. PMMA has great mechanical properties and low toxicity for the dental prosthesis fabrication (this work by Ghaith Darwish is licensed under a Creative Commons Attribution (CC BY 4.0)).

Another emergent field of application is dentistry. PMMA is a material of election for dental applications, mainly due to a its biocompatibility and aesthetics. However, it is of great importance to increase the PMMA mechanical properties used in the dental material [63]. For instance, the wear resistance of dentures based on PMMA was increased after been coated with 30 nm TiO_2_ by ALD at 65 °C [48]. A mechanical tooth-brushing device was used to assess the denture sample wear resistance and, after a brushing test, it revealed that the coating remained intact Figure 12 depicts the survey XPS spectra where the Ti peak is present in both brushed and unbrushed samples implying that the TiO_2_ thin film is stable and well adherent to the PMMA surface. Additionally, the surface microbial interactions were also studied by *Candida albicans* biofilm attachment and it was observed a reduction of microbial biofilm burden on the TiO_2_-coated PMMA surface. This result arises from surface wettability modification of the TiO_2_-coated PMMA. It is worth mentioning that, ALD technique represents a change in prothesis fabrication method that would also include a final step of TiO_2_ coating after finishing and polishing [48].

## 4. ALD Coatings on PMMA Aided by Seed Layer

The literature above presented demonstrated that PMMA is a viable polymer material for ALD coating process. However, the requirement of a low deposition temperature due to its low T*_g_* raises another limitation: the known ALD precursors for thin film formation at low temperatures are very limited. A solution can be the use of seed layers.

**Figure 13:**
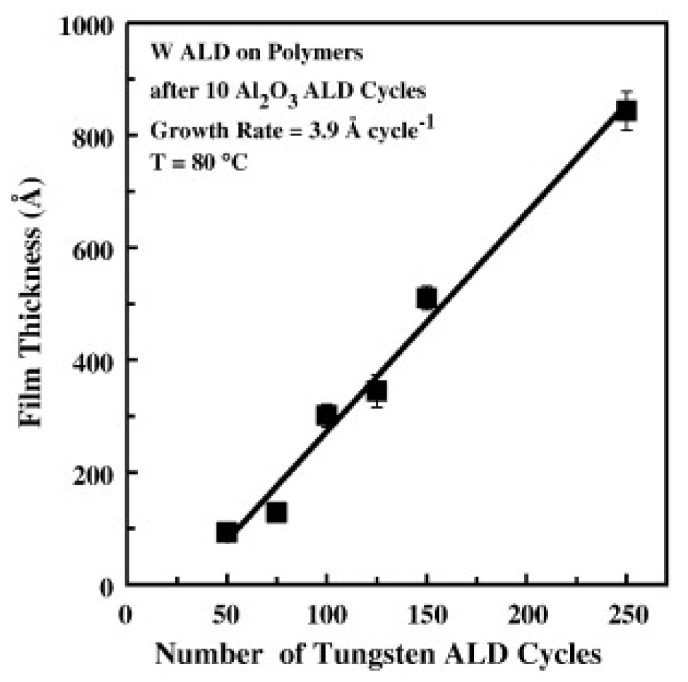
Profilometry measurements of W ALD film thickness on different polymers vs. the number of W ALD cycles. Al_2_O_3_ ALD was used as a seed layer (10 ALD cycles) (reprinted from [64], Copyright (2008), with permission from Elsevier).

Wilson et al. reported on W deposition on polymers by ALD and their results showed that W nucleation was enhanced by a few previous cycles of Al_2_O_3_ by ALD. In this case, Al_2_O_3_ acts as a seed layer of nucleation on a variety of spin-coated polymers such as PMMA, polyvinyl chloride (PVC), polystyrene (PS), polypropylene (PP), and polycarbonate (PC). A growth per cycle (GPC) of 3.9 Å for W ALD at 80 °C has been attained, as shown in Figure 13 [64].

**Figure 14:**
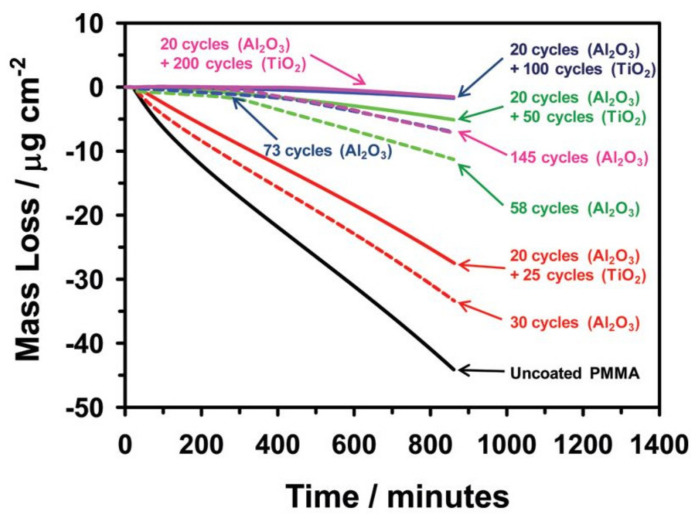
Mass loss of uncoated and coated PMMA with Al_2_O_3_ and Al_2_O_3_/TiO_2_ when exposed to vacuum UV radiation over time (reprinted with permission from [65], Copyright (2010) American Chemical Society).

Minton et al. followed the same strategy in terms of using Al_2_O_3_ ALD seed layer prior to TiO_2_ ALD, because TiO_2_ did not nucleate well on the PMMA surface. Their results showed that the uncoated PMMA lost a considerable part of its mass, when exposed in vacuum to UV radiation and the bilayers formed with 20 cycles of Al_2_O_3_ and 100 or 200 cycles of TiO_2_ were efficient in preserving PMMA (Figure 14) [65].

**Figure 15:**
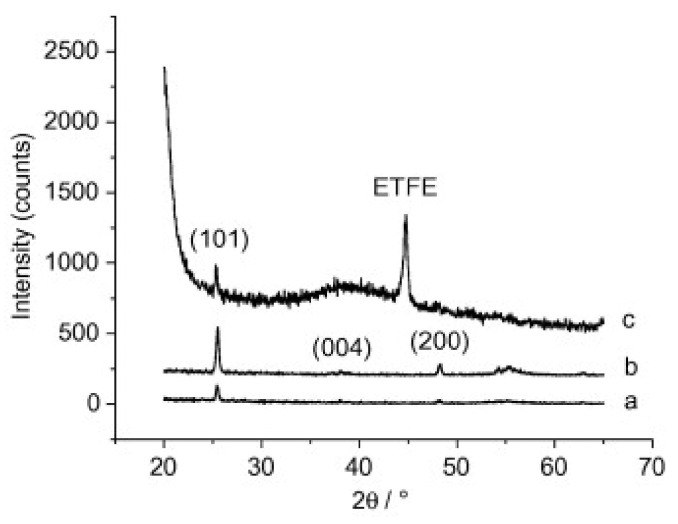
XRD patterns of anatase TiO_2_ films in substrates made of (**a**) Si at 200 °C, (**b**) PMMA at 250 °C, and (**c**) ETFE ate 200 °C. Si substrate was used as a reference, for comparison purpose (reprinted from [66] Copyright (2008), with permission from Elsevier).

Kemell et al. also explored the ability of ALD coatings of Al_2_O_3_ and TiO_2_ on PMMA films, among other polymer films, like polyether ether ketone (PEEK), polytetrafluoroethylene (PTFE), and ethylene tetrafluoroethylene (ETFE) [66]. The deposition temperature range was from 80 to 250 °C, enabling the synthesis of amorphous and crystalline metal oxides. Firstly, amorphous Al_2_O_3_ was deposited at 150–250 °C followed by TiO_2_ deposited at 100 °C. For the case of TiO_2_ deposition on Al_2_O_3_-coated PMMA at 250 °C, polycrystalline anatase TiO_2_ films were obtained, as shown in Figure 15 These results highlight the importance of Al_2_O_3_ seed layer, or interlayer, prior to TiO_2_. In fact, TiO_2_ deposition on bare PMMA was attempted also at 250 °C, but virtually no growth was observed, revealing that TiO_2_ does not nucleate well on PMMA. Interestingly, the authors did not point out any constraints of the polymer’s thermal stability when deposition at 250 °C, in particular regarding PMMA stability.

**Figure 16:**
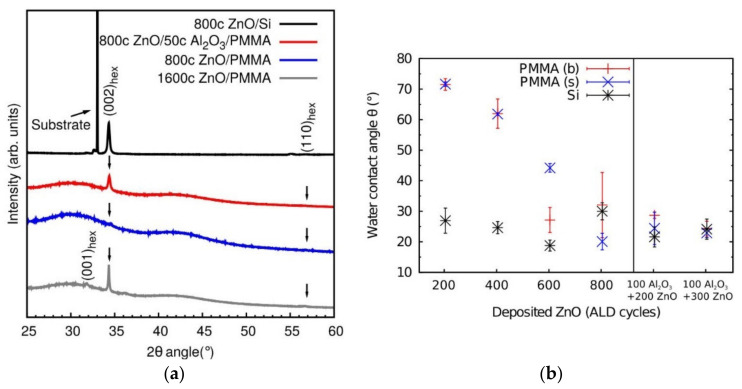
XRD patterns for 800 cycles of ZnO ALD onto: Si (black) and bulk PMMA substrates with (red) and without Al_2_O_3_ seed layer (blue), and an XRD pattern of 1600 cycles ZnO film on bulk PMMA(grey). (**a**) Water contact angles (θ) of UV-illuminated ZnO deposited on bulk PMMA (red), spin-coated PMMA (blue), and Si substrates as a function of ZnO ALD cycles (black). The two right-most points represent the contact angles of ZnO deposited on 100 cycles Al_2_O_3_ intermediate layer (**b**) (reprinted with permission from [67], Copyright (2014), American Vacuum Society).

An identical approach was performed by Napari et al. Authors reported on the influence of the deposition of an Al_2_O_3_ seed layer to the ZnO film growth, morphology, and crystallinity, on PMMA commercial plates and spin-coated PMMA films on Si substrate. The Al_2_O_3_ seed layer provides a pathway for blocking the DEZ precursor into the PMMA subsurface and improves the ZnO growth with some degree of hexagonal crystal orientation at low deposition temperature viz 35 °C (Figure 16a). The ZnO surface wetting properties were altered upon UV illumination (Figure 16b). As a consequence, this photoinduced changes on the wettability find applications in microfluidics, where thin functional coatings on patterned polymer platforms can be used to manipulate the fluid flows [67]. This work is a promising alternative for lab-on-a-chip technologies development and microfluidics platforms.

In brief, the seed layer or interlayer approach on polymers can be seen as an in-situ two-step ALD process, consisting of the deposition of a few nanometer seed-like layer, at a lower temperature step, followed by a second process for the more refractory metal oxides. The choice of Al_2_O_3_ ALD from TMA and H_2_O precursors is a viable pathway to seeding layer on polymers because Al_2_O_3_ ALD can be conducted at temperatures as low as 35 °C, conjugated with the TMA positive characteristics like its high volatility and reactivity towards different co-reactants at low temperatures. Based on the above studies, the majority of published work for ALD on polymers addresses Al_2_O_3_ ALD from TMA and H_2_O cycles, stressing out the versatility of this ALD process either as coating and/or seed layer, being also a method to study the influence of the polymer substrate properties on the nucleation and growth of metal oxides. Similar to Al_2_O_3_, ZnO ALD can also be performed on polymers by taking advantage of the DEZ high volatility and reactivity at low temperatures. It is clear that the modification of a polymer substrate that shows high reactivity towards a given ALD process is crucial to ensure high-density nucleation towards a homogeneous and uniform thin film.

## 5. Coatings on PMMA by Plasma Atomic Layer Deposition

**Figure 17:**
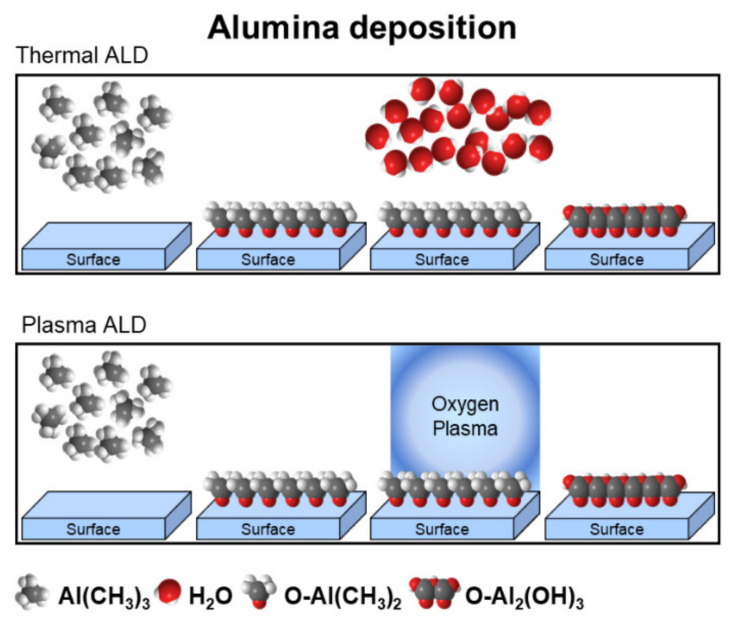
Schematic representation of one cycle of Al_2_O_3_ by thermal ALD and plasma ALD techniques. In plasma ALD, the H_2_O co-reactant is replaced with a plasma exposure (e.g., O_2_ plasma) to grow metal oxides.

Plasma-enhanced ALD (PE-ALD) is an energy enhanced ALD method. In plasma-enhanced, also referred to as plasma-assisted ALD (PA-ALD), plasma ALD simply or, in some cases, radical enhanced ALD (RE-ALD), the substrate surface is exposed to the species generated by plasma during the reactant step [68]. For instance, the synthesis of metal oxides thin films by PE-ALD is schematically illustrated in Figure 17, in which an oxygen plasma is employed during a one-step of the cyclic deposition process.

In this manner, the plasma is used to generate metastable species by gas dissociation increasing the reactivity delivered to the deposition surface. As a consequence, less thermal energy is necessary at the substrate surface to drive the ALD surface process allowing the thin film deposition at lower substrate temperatures comparing to thermal ALD [68,69]. Such high reactivity and low deposition temperatures extend the range of materials that can be used as (i) ALD precursors and (ii) thermally sensitive substrates. There are different types of plasma ALD reactor configurations: (i) in PA-ALD and PE-ALD (both meanings are the same) the substrate is exposed to the plasma discharge since it is located in the same space as the plasma source or very near the substrate; (ii) in radical enhanced ALD (RE-ALD), the plasma source is separated from the substrate so that only radicals generated by the plasma are allowed to reach the substrate [68,69,70]. The plasma is ignited from an electrical discharge from radio-frequency or microwave energy when a continuous flow of O_2_, N_2_, H_2_, or NH_3_ passes through the plasma sources [27,69]. Another aspect to take into consideration is the effect of the distance between the plasma and the sample, especially shorter distances where the substrate surface will be more exposed and more sensitive to the plasma [71,72]. The plasma ALD processes produce thin films with better characteristics, such as a lower level of impurities as a consequence of better stoichiometry, than a thin film produced by thermal ALD [17,68,73]. Nevertheless, it requires more complex equipment than that used for thermal ALD [68].

Kääriäinen et al. used non-functionalized PMMA commercial polymeric plates to deposit TiO_2_ from tetrakisdimethylamino titanium, ((CH_3_)_2_N]_4_Ti/TDMAT) and plasma excited O_2_ precursors by PA-ALD. The authors investigated the relationship between the plasma power and the carrier gas (e.g., Ar and N_2_) to improve the film adhesion on the polymeric substrates. The best result in terms of TiO_2_ film adhesion was obtained for a relatively low plasma power (25 W) with Ar carrier gas. These experimental parameters also played a role in the variation of the TiO_2_ refractive index [70].

Surface-enhanced Raman spectroscopy (SERS) has been widely used in various types of ultrasensitive sensing applications in a wide variety of fields. This analytical tool is very powerful in biosensing and material science for the detection of analytes in very low concentrations. Huebner et al. developed a PMMA-based SERS substrate to simplify the fabrication process as well as improve the biosensing response. To this end, PMMA SERS-gratings were coated with Al_2_O_3_ protective layer either by T-ALD or by PA-ALD at low deposition temperatures (80–120 °C) for both processes and no influence on their physical properties has been mentioned. Afterwards, the Al_2_O_3_-enclosed PMMA-grating was coated with thermal evaporated Ag) layer which serves as the structured plasmonic film for the enhancement of the light field [74]. It was found that a 10 nm Al_2_O_3_ ALD layer is thick enough to suppress the PMMA Raman background signal safely. Moreover, this layer is also hard and dense enough to protect the polymer against organic solvents and allows the cleaning of the SERS substrate and, thereby, repeated use for SERS measurements [74].

**Figure 18:**
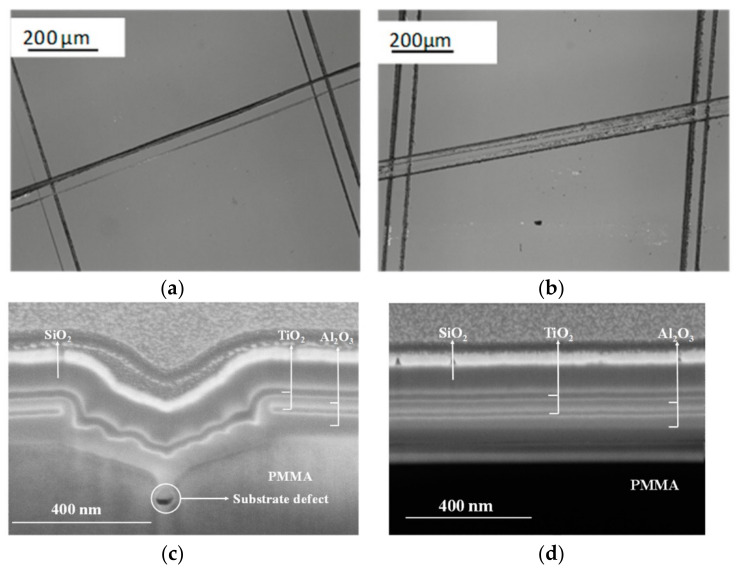
Schematic representation of one cycle of Al_2_O_3_ by thermal ALD and plasma ALD techniques. In plasma ALD, the H_2_O co-reactant is replaced with a plasma exposure (e.g., O_2_ plasma) to grow metal oxides. (**a**) Optical microscopic images after cross-hatch tests and (**b**) after climate test of antireflection coatings double-sided coated PMMA. Focused ion beam scanning electron microscopy (FIB-SEM) cross-sectional image of multilayered antireflection coatings on PMMA (**c**) focused on a crack (**d**) focused on a crack-free region (from [49], this work by Pallabi Paul is licensed under a Creative Commons Attribution (CC BY 4.0)).

Both T-ALD and PE-ALD processes were performed regarding optical components made of lightweight polymers, a good alternative to glass optics. Here, Paul et al. explored the antireflection properties of TiO_2_, Al_2_O_3_, and SiO_2_ ALD multilayered coatings on PMMA substrates. For all depositions, the temperature was kept at 60 °C which is well below the PMMA T*_g_*. After finding the ideal conditions for ALD processes, the authors conclude that the best results were achieved when an Al_2_O_3_ T-ALD layer is deposited on PMMA substrates to prevent surface cracking before the subsequent PE-ALD coatings. The plasma intensity played an important role in the film’s adhesion and refractive index towards the antireflection coating property [49]. For instance, the SiO_2_ and TiO_2_ films deposited using the ‘low’ plasma (100 W) conditions on pre-coated PMMA substrates with 40 nm Al_2_O_3_ T-ALD show no significant delamination of the film after the cross-hatch test. Figure 18 illustrates the investigated multilayered coatings on PMMA, where it is possible to discern the well-defined SiO_2_, TiO_2_, and Al_2_O_3_ layers [49].

## 6. Area Selective ALD on PMMA

### 6.1. PMMA as Masking Layer

An alternative approach for ALD on PMMA surfaces takes advantage of the low reactivity of PMMA. Several pre-treatments have been studied for PMMA surface modifications, aiming to enhance its surface hydrophilic properties. These include wet chemistry, plasma treatment and UV irradiation, where the treated PMMA either as a film or as plates are employed in biological samples immobilization and for improving component microchips [75,76,77]. However, the hydrophobic nature of PMMA is advantageous for exploiting it as inhibiting mask layers (i.e., patterned areas of the sample) to prevent ALD growth, the so-called area-selective ALD (AS-ALD), where the film is deposited only on areas without the PMMA.

Self-assembled monolayers (SAM) of PMMA can passivate the active reactions sites on the growth surface and therefore hinder ALD nucleation [51,78,79,80,81]. Färm et al. produced a patterned masking layer from a PMMA SAM to passivate the Si surface against the ALD growth of Ir, Pt, Ru, and TiO_2_ coatings. As a result, the coatings were selectively deposited on areas without the SAM [81]. TiO_2_ ALD and PMMA masked area-selective ALD approach was also explored by the research groups of Sinha [79] and Haider [51]. Cho et al. created AS-ALD using PMMA in additive and subtractive printing [82]. Also Wei et al. created a passivation hybrid with PMMA and parylene and further coating with AlO_x_ to create thin film transistors [83]. These results indicate that the PMMA films can work in area-selective ALD and the PMMA masking layer can be easily dissolved in acetone after the deposition process.

Shin et al. introduced an ALD/SAM multi-process to enhance the hydrophobic surface on PMMA, aiming at the development of antireflection coatings in self-cleaning applications. To this end, octadecyl-trichlorosilane (OTS) was chosen as SAM on PMMA, followed by Al_2_O_3_ ALD. As a result, larger water contact angle values were obtained with this multi-process when compared to those without the ALD deposition process and the SAM layer did not affect the optical transmittance properties of the coated PMMA [45].

**Figure 19:**
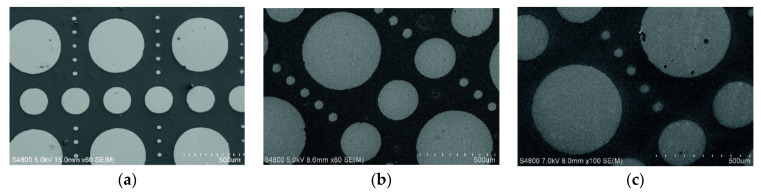

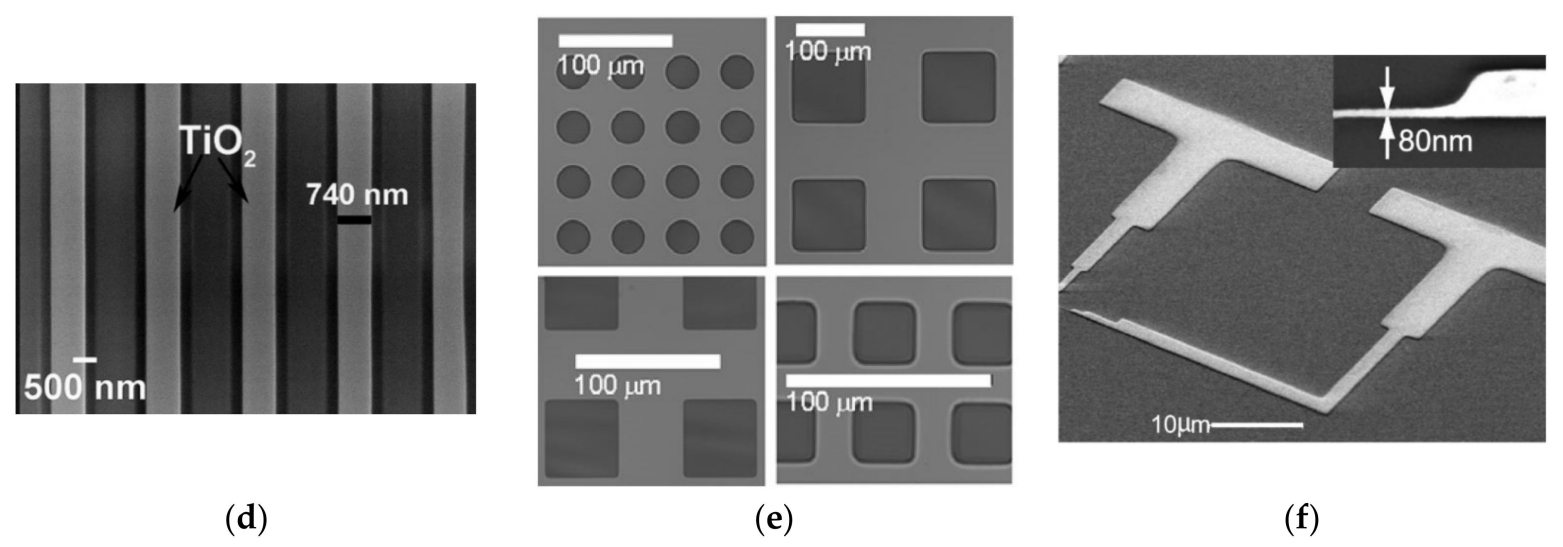
SEM micrographs of patterns produced on PMMA by AS-ALD tool for (**a**) Pt (**b**) Ir (**c**) Ru (adapted from [81], Copyright (2008), with permission from Elsevier); (**d**) TiO_2_ (adapted with permission of Ali Haider, from [97]; permission conveyed through Copyright Clearance Center, Inc.); (**e**) TiO_2_, (republished with permission of (Ashwini Sinha), from [91]; permission conveyed through Copyright Clearance Center, Inc.); (**f**) HfO_2_, (reprinted from [99] with the permission of AIP Publishing) coatings in a silicon substrate.

The PMMA removal step is an important feature in device fabrication and different strategies are ranging from wet to dry procedures. In this context, the PMMA layer can be eliminated by immersing in organic solvents (dichloromethane [84], acetone [85,86,87,88,89], isopropanol [87], or a mixture of acetone and isopropanol [87,89,90]), rinsing solvents like methanol [86,91] and finally washed with de-ionized water [25,84,91]. Sometimes, the PMMA residues are removed by annealing [86], Tan et al. and Cho et al. removed the layer with UV-ozone treatment [82,92]. Figure 19 illustrates practical examples of the various PMMA patterns in AS-ALD. These patterns are based on the following geometrical shapes: circles [91,93,94], squares [79,91,93,95,96], crosses [97], line(s) [51,82,89], and shapes or draws with more complexity [73,98]. The masking layer, resultant from the PMMA with inhibited growth, can be constituted from TiO_2_ [51,79,81,89,91,95], Al_2_O_3_ [81,82,98,99], hafnium dioxide (HfO_2_), zirconium dioxide (ZrO_2_) [99], and ZnO [73,82,97], SnO_2_ [82] Ir, Pt, and Ru [81].

The PMMA mask can also be processed as a patterned layer, the first step consisting in spin-coating the polymer over the substrate [73,79,91,93,95,96,97,98,100,101,102,103] and, sometimes, heat treating at 180 °C [51,93,97,99] or soft heat treating [51,79,91,96] for a short time, followed by an etching process to reveal the desired pattern. The techniques employed to etch the PMMA substrate and create the pattern are: (1) lithography (optical lithography [96], photolithography [93,97], deep-UV lithography [79], electron beam lithography [51,85,89,100,103]); (2) heated cantilever probe tip (thermal writing) [95]; (3) chemical writing with isopropanol: methyl isobutyl ketone: methyl ethyl ketone in 75:24:1 ratios [99]; (4) nanoimprint and etching [98,101].

After the patterning, it is necessary to create a smooth surface and clean the excess of PMMA to regularize the template shapes; some authors suggest the O_2_ plasma descum etch [85,95,101] while others advise vacuum annealing [79,91]. Färm et al. and Sinha et al. used a similar approach exposing the resultant pattern to a solution of isopropyl alcohol/methyl isopropyl ketone [81] or isopropyl alcohol/methyl isobutyl ketone [79]. Both rinsed the material with isopropyl alcohol and water and pre-dried with a nitrogen stream; the last step consisted of drying it in an oven at 100 °C [79,81]. A similar method uses methylisobutylketon: isopropyl alcohol solution and rinsing with the same alcohol [93]. Other authors simply did the ALD after the patterning [91,97,103]. Following this, the ALD takes place over the total material area—i.e., substrate and the PMMA pattern. Sharma et al. lift-off the PMMA with acetone [103]. Färm et al. and Sinha et al. used an ultrasonic bath with acetone, to ensure the PMMA removal. However, the total processes took about 1 h [79,81,91]. Tang et al. and Dhuey et al. hastened this process by substituting the acetone dipping for O_2_ plasma [98,100].

### 6.2. ALD on Di-Block Copolymer Masks

**Figure 20:**
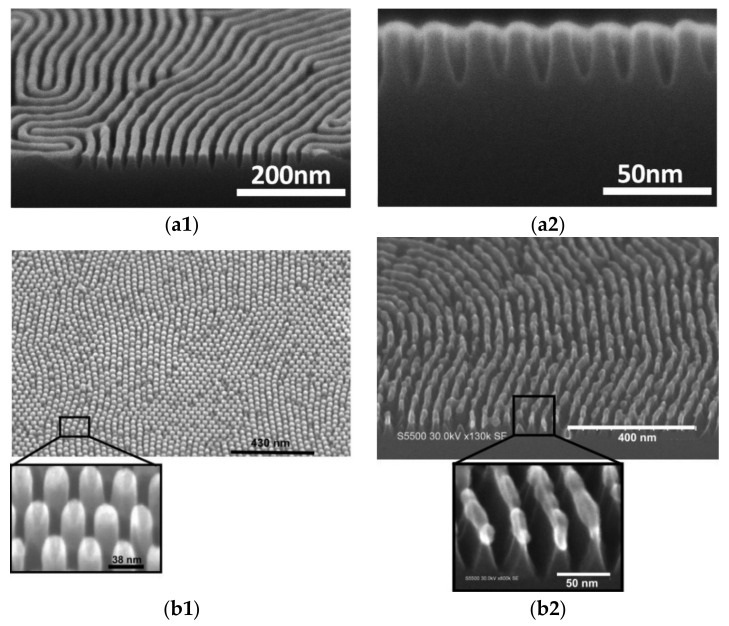
SEM micrographs of patterns produced from a diblock copolymer with PMMA; (**a1**) cross-section top view (**a2**) of Si nanowire array with an Al_2_O_3_ mask (adapted from [80], Copyright (2015), with permission from Elsevier). Top view, 40° tilt on top and cross-section of (**b1**) Al_2_O_3_ hexagonally packed nanopillars array treated with plasma and (**b2**) nanowires made of Si with Al_2_O_3_ mask on top (adapted and republished with permission of Guillaume Gay, from [106]; permission conveyed through Copyright Clearance Center, Inc.).

Another approach for AS-ALD consists in using block copolymer (BCP) layer(s) to generate nm-sized features—e.g., nanotemplates [78,80,104] (Figure 20). The strategy is to choose a polymer that delays the nucleation or does not promote any nucleation, such as polystyrene in PS-*b*-PMMA [78,80,104,105,106], polystyrene-r-poly(methyl methacrylate) (PS-*r*-PMMA) [78,106,107], and poly(styrene-co-methylmethacrylate-co-hydroxyethyl methacrylate) [80].

The route starts with the deposition of the BCP in substrates (silicon wafer [78,80,105,106] or magnesium oxide [104]) by spin-coating. Subsequently, BCP is annealed at a temperature above the glass transition temperature of both polymers [80] to neutralize and remove the excess solvent, followed by cleaning to eliminate the non-anchored chains [78,80,106]. Some authors add another BCP to create another layer, and the first one is named as brush layer; after the BCP deposition, the whole structure is annealed. Both layers have the same interfacial tension.

Hence, after annealing, the BCP film assembled into PMMA lamellae or cylinders. The transformation is spontaneous, resulting in a pattern with high aspect ratio and sharp edge [107]. Peng et al. experimented with just one BCP, where the ALD deposition happens over the pattern and, finally, the sample is treated with O_2_ to remove the polymers and cleaned to ensure the total polymeric elimination [105]. For templates with a brush layer, there is no defined order for the subsequent steps. In fact, the desired effect depends on the order: (1) ALD deposition over the template; (2) exposition to UV light [78], piranha solution [80], plasma [105], acetic acid [78,106] or etching [104] to remove the PMMA and PS from the DBC and the brush layer; (3) cleaning to remove excess material from (2). According to the desired method, there is a possibility to remove the unexposed parts of the substrate by etching [80,106].

**Figure 21:**
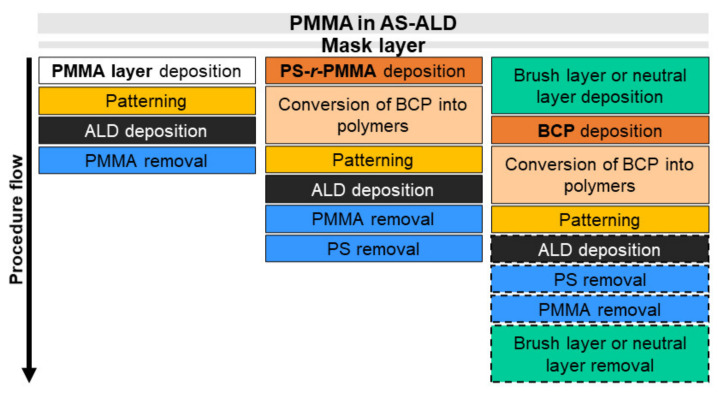
PMMA in an AS-ALD process, where the block copolymers are polystyrene-*random-*poly(methylmethacrylate) (PS-*r*-PMMA) or polystyrene-*block*-poly(methylmethacrylate) (PS-*b*-PMMA).

In resume, PMMA has proven to be a versatile polymer material, in a wide range of applications and is often chosen as a processing layer for AS-ALD of pure metals and metal oxides. Figure 21 illustrates the various PMMA material forms and processing stages in an AS-ALD process.

## 7. Summary and Prospective

Table 1 is a compilation of the main details of the supporting literature of this review devoted to the modification of PMMA surfaces by ALD technique. The table is organized by ALD types (thermal, plasma, and area-selective) on different PMMA substrate geometries and describes the mainly ALD experimental details, such as deposition temperature, types of precursors, and film thickness. Moreover, the table points out the different research topics of each paper, ranging from fundamental knowledge to practical applications.

A significant number of systems have been developed, which are mostly based on thermal or plasma-enhanced ALD. Naturally, both of these techniques have benefits and drawbacks to assure a substrate compatible process, as well as a dense and uniform coating. The substrate sensitivity determines the ALD processing technique and respective parameters. A low deposition temperature is preferred when the substrate is heat-sensitive and/or energy consumption is a major concern in industrial production. The most well established ALD processes involve the deposition of inorganic metal oxides on Si wafers. However, the reviewed literature shows continuous efforts to expand the ALD processes to the surface modification of polymers, particularly PMMA. Consequently, PMMA will improve and/or add new functionalities to our daily materials and give a unique opportunity to develop add-value products.

The thermal fragility of the PMMA polymer as a substrate requires the deposition temperature of a film on its surface to be below of the glass transition temperature of PMMA (55–130 °C). Despite this limitation, Al_2_O_3_ ZnO and TiO_2_ thin films have been successfully deposited onto PMMA substrates by low temperature (T < 100 °C) ALD processes, which are effective active layers or masking layers. Moreover, the ALD method is very sensitive to surface chemistry and therefore offers an ideal solution for continuous or patterned thin films. The use of PMMA polymer films for area selective atomic layer deposition (AS-ALD) has thus brought extra advantages to create the micro and nanopatterning due to their facile removal after the selective deposition process is completed.

More broadly, the achievement of ALD thin film formation on a polymer surface opens doors in the field of functional organic–inorganic interfaces, evolving advanced nanofabrication techniques that will provide opportunities for new levels of materials and their miniaturization with exceptional properties.

## Figures and Tables

**Table 1:** Summary of literature data regarding ALD coatings on PMMA.

ALD Type	PMMA Substrate Geometry	Precursors, Time Sequence (Pulse/Purge/Pulse/Purge Times)	Number of Cycles (Film Thickness)	T_Deposition_	Application or Motivation	**Ref**
Thermal ALD on PMMA	Film (~70 nm) M_W_ = 350.000 spin-coated onto polished Si (100) ^a^	**ZnO** DEZ/N_2_/H_2_O/N_2_	1000 (25 nm)	25 °C	Curved organic light emitting diodes	[108]
	1 mm sheet and film (2–2.5 μm) M_W_ = 950 kDa spin-coated onto Si(111)	**ZnO** DEZ/N_2_/H_2_O/N_2_ 0.15–0.30/30/0.15–0.30/30 s **Al_2_O_3_** TMA/N_2_/ H_2_O/N_2_ 0.30/30/0.3/30 s	100–800 (30 nm grains) 20–100 (10 nm) Al_2_O_3_ + ZnO 20–50 + 200–800	35 °C	Microfluidics	[67]
	Film (5, 32, and 80 nm) M_W_ = 350.000 spin-coated onto Si(100) ^b^	**ZnO** DEZ/N_2_/ H_2_O/N_2_ 1/120/1/120 s	150 (21.2, 18.6, 15.9 nm)	35 °C	Flexible electronics and nanoscale devices	[58]
	Plates 2 mm M_W_ = 150 000–160 000	**TiO_2_** TDMAT, 40 °C/N_2_/O_3_/N_2_ 0.5/10/4/10 s **Al_2_O_3_** TMA, 20 °C/N_2_/O_3_/N_2_ 0.25/6/4/6 s	1000 (60 nm) 1000 (85 nm)	60 to 65 °C	Nanoindentation and nanotribology studies	[53]
	Sheet extruded	**Al_2_O_3_** TMA/residence/N_2_/H_2_O/residence/N_2_ 0.10/10/45/0.2/10/45 s	750 (130 nm)	65 °C	To increase interfacial toughness	[54]
	Specimens (20 × 20 × 1 mm)	**TiO_2_** TDMAT, 65 °C/Ar/O_3_/Ar 0.5/10/1/15 s	300 (30 nm)	65 °C	Dental implants	[48]
	PMMA NPs (50-100 nm)	**TiO_2_** TTIP/purge/H_2_O/purge 0.5/15/0.5/15 s	250	80 °C	Photocatalysis	[59]
	Film (~200 nm) M_W_ = 350.000 spin-coated onto Si wafer ^c^	**Al_2_O_3_** TMA/Ar/H_2_O/Ar 60/30/60/30 s 5/60/5/60 s	(200 nm)	80 °C	Study of the ALD mechanisms	[52]
	Particles (∼1–100 μm) and film M_W_ = 15.000 spin-coated onto silicon substrates	**W** WF_6_/N_2_/Si_2_H_6_/N_2_ 1/60/5/60 s **Al_2_O_3_** TMA/N_2_/H_2_O/N_2_ 1/60/5/60 s	Film: Al_2_O_3_ + W 10 + 50–250 (95–845 Å) Particles: W 25-200Al_2_O_3_ + W 5 + 25 (29 Å)	80 °C	Flexible optical mirrors, electromagnetic interference shielding, diffusion barriers	[64]
	Powder (0.2–1 mm) M_W_ = 120 kDa	**ZnO** DEZ, 22 °C/N_2_/H_2_O/N_2_ 0.3/3/0.1/5 s	400 (80 nm)	80 °C	Photocatalysis	[62]
	Plates (4 mm) and powder (0.2–1 mm) M_W_ = 120 kDa	**ZnO** DEZ, 22 °C/N_2_/H_2_O/N_2_ 0.3/3/0.1/5 s	Plates: 220–2200 (1–180nm) 1650 (100 nm) Powder: 1650 (80 nm)	80 °C	Photocatalysis	[60]
	Flat (2.5 × 2.5 cm) ^d^	**ZnO** DEZ, 22 °C/N_2_/H_2_O/N_2_ 0.3/3/0.1/5 s	1650 (100 nm)	80 °C	Water reuse	[61]
Thermal ALD ALD on PMMA (cont.)	Film (4000 ± 1000 Å) M_W_ = 15.000 spin-coated onto Si(100) wafer and QMC sensors	**Al_2_O_3_** TMA/N_2_/H_2_O/N_2_ 1/29/1/29 s	30 cycles (1000–1500 Å)	85 °C	Organic light emitting diode	[40]
	Film M_W_ = 15.000 spin-coated onto QCM Discs ∼2 µm	**TiO_2_** TiCl_4_/N_2_/H_2_O/N_2_ **Al_2_O_3_** TMA/N_2_/H_2_O/N_2_ 2/30/2/30 s	Al_2_O_3_ 30–45 (23.42–51.89 Å) Al_2_O_3_ + TiO_2_ 20 + 25–200 (26.09–146.84 Å)	90 °C	Aerospacial	[65]
	Film (305 nm) M_W_ = 120.000 with β-NaYF_4_:Er^+3^ NPs spin-coated onto borofloat 33 glass	**TiO_2_** TiCl_4_/Ar/H_2_O/Ar	(199 nm total)	100 °C	Upconversion luminescence	[56,57]
	(1.5 × 1.5 cm)	**TiO_2_** TDMAT, 70 °C /N_2_/O_3_/ N_2_ 1/15/1.8/15 s	50–500 (75–425 Å)	120 °C	Wettability and hardness improvement	[55]
	Flat (2 × 2 cm^2^) ^d^ pre-treated with OTS and heptane solution (0.1:136), at 60 °C (5–30 min)	**Al_2_O_3_**	300–1200 (9–26 nm)	150 °C	Wettability improvement	[45]
	Film (0.1 μm) spin-coated onto (100) Y-stabilized ZrO_2_ single crystal	**CeO_2_** (Ce(thd)_4_ and O_3_)	200 (3.5–5.5 nm)	200 °C	Memories technology	[50]
	Film (70–100 nm) M_W_ = 350.000 spin-coated onto Si	**TiO_2_** TiCl_4_/N_2_/H_2_O/N_2_ 0.2 s/30 s/0.2 s/30 s **Al_2_O_3_** TMA/N_2_/H_2_O/N_2_ 0.2 s/4 s/ 0.2 s/ 4 s AlCl_3_/N_2_/H_2_O/N_2_ 0.5 s/2 s/1 s/2 s **Al_2_O_3_** (TMA) + TiO_2_ 0.2/4/0.2/4 s + 0.2/4/0.2/ 4 s	700 (20 nm) 3300 (350 nm) 1000 (100 nm) 200 + 1000 (43 nm)	100 °C 250 °C 250 °C 250 °C	Wettability improvement	[66]
Plasma ALD on PMMA		**O_2_ Plasma 1** (300 W,50 sccm) **O_2_ Plasma 2** (100W, 90 sccm) **Al_2_O_3_** TMA/purge/Plasma 2/purge 0.2/10/5/5 s **SiO_2_** 3DMAS/residence/purge/Plasmas/purge 0.4/4/10/3/6 s **TiO_2_** TTIP/purge/Plasmas/purge 1.5/7/6/5 s	(80 nm) (40 nm) (55 nm)	60 °C	Antireflection coatings	[49]
Plasma ALD (cont.)	PLEXIGLAS^®^ XT Extruded acrylic sheets M_W_ = 150.000–160.000	Plasma (25–200 W) **TiO_2_** TDMAT/N_2_/plasma/N_2_ 0.5/10/0.25–6/10 s	500	50 to 70 °C	Adhesion improvement	[70]
	Film (100 nm) on quartz	**Al_2_O_3_** (TMA and O_2_ plasma)	(10 nm)	80 to 120 °C	Substrates for SERS	[74]
Area selective ALD on PMMA	Lines (10–15 nm) by electron beam lithography	**Al_2_O_3_** TMA/purge/O_2_ plasma 30/60/210 ms	30 (6 nm)	25 °C	Fabrication of high-resolution imprint templates	[100]
	Squares by electron beam lithograph	**O_2_ Plasma** (100 W) **MoO_x_** [(N^t^Bu)_2_(NMe_2_)_2_Mo]/Ar/Plasma/Ar 6/6/8/6 s	10–60 (1–4.5 nm)	50 °C	Nano and Optoelectronic applications	[103]
	Nanoporous film (75,000 g/mol) by electron beam lithography spin-coated onto Si/SiO_2_	**ZnO** DEZ/ N_2_/H_2_O/N_2_ 0.3/2/0.3/2 s	25–225 (4–26 nm)	70 °C	Fabrication of charge-trap flash memories components	[101]
	Film ~350 nm (200 and 950 k) spin-coated onto Si/SiO_2_	**Al_2_O_3_** (TMA/N_2_/H_2_O/N_2_) **HfO_2_** (TDMAH/N_2_/H_2_O/N_2_) **ZrO_2_** (TDMAz /N_2_/H_2_O/N_2_)	(2.5–50 nm) (10–25 nm) (25–100 nm)	100 to 150 °C	Microelectronics and nanoelectronics	[99]
	Stripe (312 nm) M_W_ = 950.000 by etching spin-coated onto SiO_2_/Si	**ZnO** DEZ/Ar/H_2_O/Ar 0.05/45/0.1/45 s **Al_2_O_3_** DMAI/Ar/ H_2_O/Ar 0.05/45/0.1/45 s **SnO_2_** TDMASn/ Ar/ H_2_O/Ar 0.15/45/0.1/45 s	5–30 supercycles ratio 6:5 zinc/tin 1:1 zinc/tin 10:1 zinc/aluminum 15:1 zinc/aluminum	100 to 170 °C	Fabrication of bottom-gate, top-contact thin-films for transistors	[82]
	Stripes 300 nm (950k) by electron beam lithography spin-coated onto SiO_2_/Si	**TiO_2_** TiCl_4_/N_2_/H_2_O/N_2_ 0.1/10/3/10 s	25–300 (0–14 nm)	120 °C	Photocatalysis	[89]
	Film (110 nm) M_W_ = 15.000 spin-coated onto silicon wafer	**TiO_2_** TTIP/N_2_/H_2_O/N_2_ 5/30/5/30 s	200 (2 nm)	140 °C	Heat cantilever probes	[95]
	Stripes from a ≈43 nm film M_W_ = 350.000 spin-coated onto Si(100) pre-treated with O_2_ plasma(2 min)	**TiO_2_** TDMAT/N_2_/H_2_O/N_2_ 0.03/20/0.015/20 s	100–1200 (43–23.96 nm)	150 °C	Inhibition efficacy of TiO_2_	[51]
	Squares from a (32–420 nm) film M_W_ = 54.000 coated onto Si wafer ^e^	**TiO_2_** TTIP, 82 °C/N_2_/H_2_O/ N_2_ 2/25/1/60 s **TiCl_4_,** 25°C /N_2_/H_2_O/N_2_ 2/25/2/30 s	150 (~10.5 nm) 500 (35 nm)	140 °C 160 °C	Amplified photoresist polymers	[91]
	Squares from a (32–420 nm) film M_W_ = 54.000 coated onto Si wafer ^e^	**TiO_2_** TiCl_4_/N_2_/H_2_O/N_2_ 2/25/1/60 s	150	160 °C	Amplified photoresist polymers	[79]
Area selective ALD on PMMA (cont.)	Squares from a 100 nm M_W_ = 54.000 film spin-coated onto p-type Si(100) ^f^	**TiO_2_** TiCl_4_, 23 °C/N_2_/H_2_O/N_2_ TTIP, 85 °C/N_2_/H_2_O/N_2_ 2/60/2/60 s	50–400 (3.5–28 nm) 50–500 (3.4–35 nm)	160 °C	Comparison of precursors	[109]
	Stripes from a (9–40 Å) film spin-coated onto Si wafer pre-treated with O_2_ plasma	**ZnO** DEZ/63 ms/purge/H_2_O/63 ms/purge	600 (40 Å)	200 °C	Thin-film transistors	[97]
	Dots (50–500 nm of diameter) from a film (70–100 nm) M_W_ = 350.000 spin-coated onto Si (100)	**TiO_2_** (Ti(OMe)_4_ and H_2_O) **Ru** (RuCp_2_ and air) **Pt** (MeCpPtMe_3_ and O_2_) **Ir** (Ir(acac)_3_ and O_2_) **Al_2_O_3_** (AlCl_3_ and H_2_O) **Al_2_O_3_** (TMA and H_2_O)	500 500–100 2700 1000–500 500 (40 nm) 500	250 to 300 °C	Passivation effect studies	[102]
	Squares or circles from a film spin-coated onto Si (100) wafer	**Pt** (MeCpPtMe_3_ e O_2_)	1000 (50.4 nm)	300 °C	Nanotechnology	[93]
Area selective ALD on Diblock Copolymers	PMMA cylinders (diameter 30 ± 3 nm) from treated PS-*b*-PMMA (60 nm), previously spin-coated onto SiO_2_/Si	**Al_2_O_3_** TMA, 25 °C/N_2_/H_2_O, 25 °C/N_2_ 60/300/60/300 s 300/300/300/300 s **TiO_2_** TiCl_4_, 25 °C/N_2_/H_2_O, 25 °C/N_2_ 300 s/300 s/300 s/300 s	10 (8.48 nm) 10 (30.8 nm) 5–10 (13.3–16.9 nm)	85 °C 85 °C 135 °C	Molecular sensing	[105]
	PMMA blocks from treated PS-*b*-PMMA (25 nm), previously spin-coated onto (8 nm) SiO_2_/Si	**Al_2_O_3_** TMA/purge/H_2_O/purge 30/60/30/60 s	10 (14.3 nm)	130 °C	Nanofabrication for complementary metal oxide semiconductor technology	[80]
	PMMA hexagonal nanopores from treated Ps-*r*-PMMA and PS-*b*-PMMA, previously spin-coated in SiO_2_/Si(100) wafers (50 nm) ^g^	**Al_2_O_3_** TMA/N_2_/H_2_O/ N_2_ 0.2 s/8 s/ 0.2 s/10 s	22 (2.2 ± 0.1 nm) 122 (10.7 ± 0.1 nm)	300 °C	Biomedical devices	[78]

Cleaning methods: ^a,b,d,g^ piranha solution; **^c^** JTP cleaning; ^e,f^ 2M HNO_3_ for 2 h; 3DMAS, Tris(dimethylamino)silane ((Me_2_N)3SiH); AlCl_3_, Aluminum trichloride; Ce(thd)_4_, Tetrakis(2,2,6,6-tetramethyl-3,5-heptanedionato)cerium (Ce(C_11_H_19_O_2_)_4_); CeO_2_, Ceric dioxide; HfO_2_, Hafnium dioxide; Ir(acac)_3_, Iridium acetylacetonate; MeCpPtMe_3_, [(N^t^Bu)_2_(NMe_2_)_2_Mo], bis(tertbutylimido)-bis(dimethylamido)molybdenum; Trimethyl(methylcyclopentadienyl)platinum(IV); OTS, trichloro(octadecyl)silane; RuCp_2_, Ruthernium dicyclopentadienyl; SERS, surface-enhanced Raman spectroscopy; Si_2_H_6_, Disilane; TDMAH, Tetrakis(dimethylamido)hafnium ([(CH3)_2_N]_4_Hf); TDMASn, Tetrakis(dimethylamido)tin ([(CH_3_)_2_N]_4_Sn]) TDMAz, Tetrakis(dimethylamido)zirconium ([(CH_3_)_2_N]_4_Zr); Ti(OMe)_4_, Titanium tretamethoxide; TiCl_4_, Titanium tetrachloride; TTIP, Titanium tetraisopropoxide (Ti[OCH(CH_3_)_2_]_4_); WF_6_, Tungsten hexafluoride; ZrO_2_, Zirconium dioxide; β-NaYF_4_:25%Er^+3^_,_ sodium yttrium fluoride doped with trivalent erbium ions.

## Data Availability

The data presented in this study are available on request from the corresponding author.
